# Bioengineering considerations in liver regenerative medicine

**DOI:** 10.1186/s13036-017-0081-4

**Published:** 2017-11-26

**Authors:** Ogechi Ogoke, Janet Oluwole, Natesh Parashurama

**Affiliations:** 1 Department of Chemical and Biological Engineering, University at Buffalo (State University of New York), Furnas Hall, Buffalo, NY 14260 USA; 2 Clinical and Translation Research Center (CTRC), University at Buffalo (State University of New York), 875 Ellicott St., Buffalo, NY 14203 USA; 3 Department of Biomedical Engineering, University at Buffalo (State University of New York), Furnas Hall, 907 Furnas Hall, Buffalo, NY 14260 USA

**Keywords:** Liver transplantation, Liver regeneration, Primary hepatocyte cell culture, Bioartificial liver, Hepatocyte transplantation, Liver cell therapies, Mouse liver repopulation, Liver cell therapies, Adult liver stem cell/progenitor cells, Pluripotent stem cells, Hepatoxicity and engineered devices, Decellularized liver grafts

## Abstract

**Background:**

Liver disease contributes significantly to global disease burden and is associated with rising incidence and escalating costs. It is likely that innovative approaches, arising from the emerging field of liver regenerative medicine, will counter these trends.

**Main body:**

Liver regenerative medicine is a rapidly expanding field based on a rich history of basic investigations into the nature of liver structure, physiology, development, regeneration, and function. With a bioengineering perspective, we discuss all major subfields within liver regenerative medicine, focusing on the history, seminal publications, recent progress within these fields, and commercialization efforts. The areas reviewed include fundamental aspects of liver transplantation, liver regeneration, primary hepatocyte cell culture, bioartificial liver, hepatocyte transplantation and liver cell therapies, mouse liver repopulation, adult liver stem cell/progenitor cells, pluripotent stem cells, hepatic microdevices, and decellularized liver grafts.

**Conclusion:**

These studies highlight the creative directions of liver regenerative medicine, the collective efforts of scientists, engineers, and doctors, and the bright outlook for a wide range of approaches and applications which will impact patients with liver disease.

## Background

### The increasing global burden of liver disease

The incidence and prevalence of chronic liver disease (CLD), manifested by the presence of fibrosis/cirrhosis and end stage liver disease, is reaching epidemic proportions worldwide, with 50 million affected. In developed countries, like the US, UK, Spain, and France, CLD rates have risen such that it is a leading cause of death (UK national statistics, https://www.gov.uk/government/statistics). In the US, more than 5 million Americans are living with CLD and by 2020, cirrhosis is projected to be the 12th leading cause of mortality [1]. The increased prevalence of CLD is linked to several factors, including non-alcoholic fatty liver disease (NAFLD) and associated nonalcoholic steatohepatitis (NASH) [2], Hepatitis B and C [3], and alcoholic hepatitis [4]. Furthermore, hepatocellular carcinoma (HCC), one of the leading causes of death worldwide, is rapidly increasing in incidence, and advanced HCC is treated with liver transplantation, and is thus relevant to liver regenerative medicine [5].

### Liver functions and liver mass

**Fig. 1:**
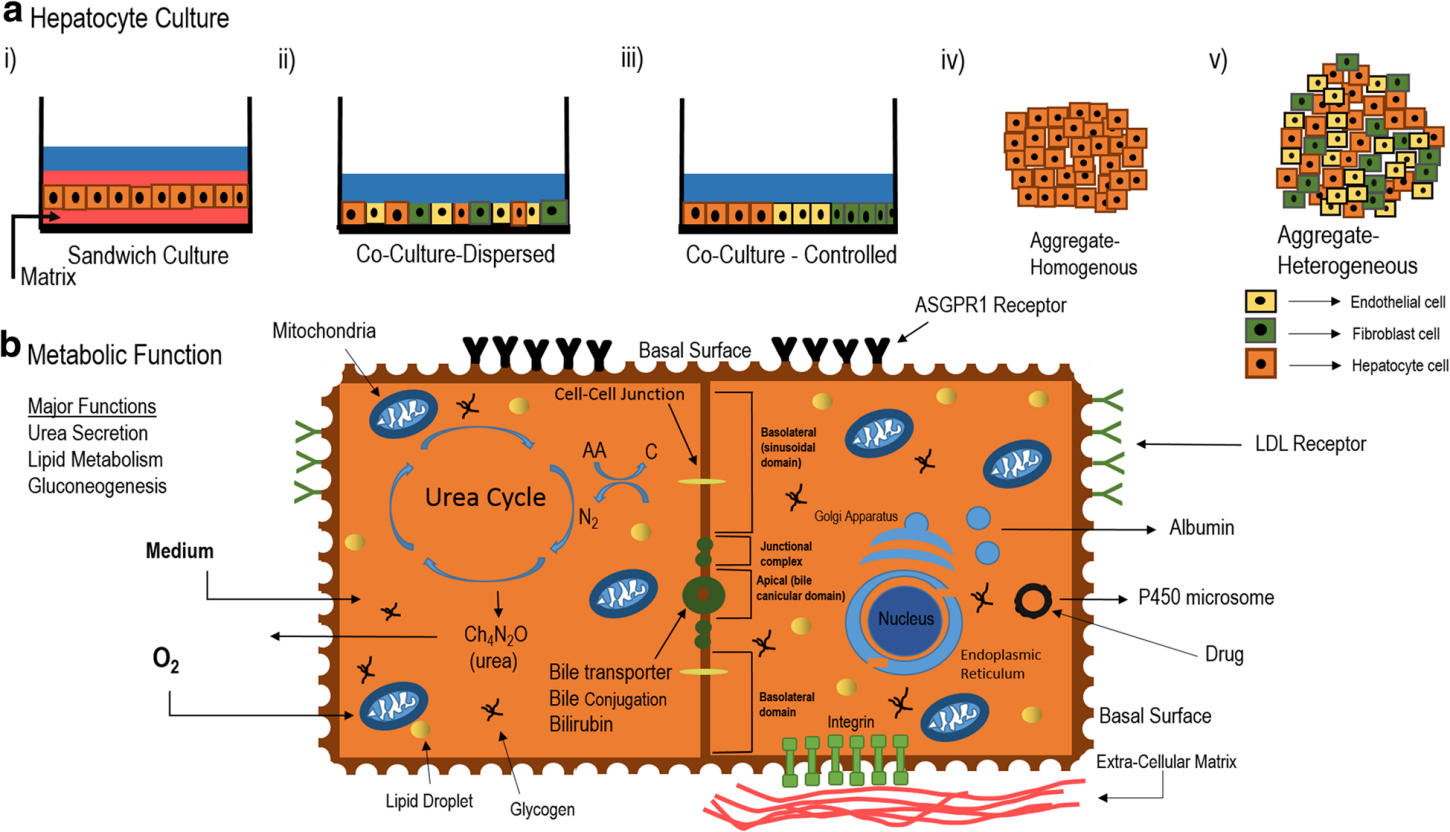
Hepatocyte culture and functions. **a** Hepatocyte culture configurations are critical to modeling in vitro functions. Several techniques are known to support not only increased levels of liver-specific gene expression, but also metabolic and physiological functions in long term culture. i) Sandwich culture provides long term physiological morphology and function and maintains epithelial structure and lateral, basal, and apical membrane domains. ii) Heterogeneous cell co-culture provides critical cell-cell heterotypic interactions between hepatocytes and supporting cells, like NIH 3T3-J2 fibroblasts that represent stellate cells and endothelial cells that represent liver sinusoidal endothelial cells, which together promotes liver functions. iii) Same as ii) except controlled cell co-culture, often using selective cell adhesion, micropatterning and microfabrication technology. iv) Liver cell aggregate culture (homogenous) enhances cell-cell contacts compared to cell matrix contacts and promotes liver function. v) Same as iv) except heterogeneous aggregate containing multiple supporting cell types that promote heterotypic cell-cell contacts. **b** Hepatocyte functions in culture. The liver is responsible for a number of important physiological and biochemical functions that can be analyzed within in vitro cultures. We depict two hepatocytes with preserved cell-cell junctional complexes, and membrane domains, including the basal, lateral, baso-lateral, and apical (bile canalicular) domains. The hepatocyte on the left demonstrates various metabolic activities of the liver, including protein, fat and carbohydrate metabolism. Glycogen storage, glycogenolysis, and gluconeogenesis refer to different metabolic processes for regulating whole body glucose levels, as well as the uptake and release of glucose for cellular metabolism. Lipids are also oxidized in the liver, and triglycerides are metabolized to produce energy. Lipoproteins, are also synthesized in the liver. Further, the liver regulates the deamination and transamination of amino acids (AA) into carbon skeletons and also regulates the removal of ammonia (N2) by urea synthesis. The liver contains many mitochondria that reduce oxygen and generate cellular energy via the electron transport chain. The liver has many other functions not shown. The cellular medium is critical, and must contain hormones, and growth factors that support these functions. The hepatocyte to the right depicts key hepatocellular functions like the synthesis and secretion of albumin, the expression of P450 microsomal enzymes for drug metabolism, expression of low density lipoprotein receptor (LDL), the expression of asialoglycoprotein receptor (ASGPR) for clearing asialyated proteins, and the expression of integrins for engaging extracellular matrix, particularly collagen Type IV in the basement membrane. The liver also synthesizes a majority of the clotting factors needed in blood coagulation

The liver is the largest internal organ and bears the unique ability to regenerate itself, whilst performing central metabolic, detoxification, synthetic, digestive, endocrine, immunoregulatory, and exocrine functions (Fig. 1). The parenchymal cell of the liver, the hepatocyte, is a complex, energetically intensive, polarized epithelial cell. The mass of the liver is central to its function.

The human adult liver weighs approximately 1.4–1.7 kg, with a hepatocyte density of 1.1–1.6 × 10^8^ cells /g [6], and has an estimated number of 2 × 10^11^ or 200 billion hepatocytes. A 7–10 week old Sprague-Dawley adult female rat, with a weight of 150–200 g and liver weight of about 7.7 g, bears approximately 1 × 10^9^ or 1 billion hepatocytes [6]. An adult 8–10 week old BL6 adult female mouse, with a weight of 16 g and a liver weight of 1.25 g, bears approximately 1.5 × 10^8^ or 150 million hepatocytes [6]. A key point is that replacing about 10% of liver function, which is still a large mass of cells, can support liver function in acute disease [7].

### Liver transplantation

Despite the increasing need for liver tissue and a shortage of available donor livers, the current standard of care for end stage liver disease is liver transplantation. Approximately 20,000 orthotopic liver transplants are conducted annually worldwide. In the US, the 1 year survival rate is 85%–88%, the 5-year survival rate is 74%, and 2000 patients die annually waiting for a donor liver [8]. Orthotopic liver transplantation was aggressively investigated following successful transplantation of the bone marrow and the kidney in the mid 1950’s [9]. Years of basic and preclinical research led to reduced complications and increased survival rates, such that currently, the most common risk factor resulting in liver failure post-transplantation is poor quality of the donor liver. Scarcity of healthy donor livers is being addressed by increasing the donor pool, improving preservation of the graft, and minimizing time of post-harvest survival prior to transplant. Machine perfusion of the donor liver is a promising approach for increasing the donor pool [10]. This approach can function either by reducing complications associated with traditional storage and transplantation, or by metabolic reconditioning livers which are considered marginal. Living donor liver transplantation introduces alternate techniques for harvesting of living liver tissue followed by transplantation, without the need for cryopreservation with subsequent liver ischemia. This approach is particularly relevant for pediatric patients, due to limited donor size for living donors [11]. Collectively, liver transplantation is successful because technical and scientific aspects have been addressed, but the procedures themselves are expensive, and patients require lifelong immunosuppression.

### Liver regeneration

Liver regeneration stemming from the loss of hepatic tissue due to injury is a unique property among the internal organs [12, 13]. The hepatocyte is the parenchymal cell of the liver, and is mature, quiescent, and expresses a vast array of differentiated genes to support its extensive functions (Fig. 1). In spite of its mature differentiated functions, the hepatocyte cell cycle is activated in response to acute liver injury, such as two thirds hepatectomy. During liver regeneration, synchronized replication of hepatocytes [14], followed by coordinated replication of nonparenchymal cells, leads to rapid and complete replacement of liver mass, function, and microarchitecture. Importantly, during regeneration, hepatocytes express over 1000 genes while sustaining essential liver functions that ensure survival of the organism [13]. Despite this robust regenerative response in acute disease, regeneration is dysfunctional in cirrhosis, and in this case, does not appreciably restore normal hepatic tissue homeostasis, microarchitecture and function.

### Early studies in liver regenerative medicine

The roots of modern liver regenerative medicine began in part with development of an apparatus to study the basic science and biochemistry of isolated perfused rat livers [15, 16]. Here, the whole liver can be removed from the intact organism, bathed, maintained to improve viability, and perfused under pseudo-physiological conditions. These techniques, together with improved analytical capabilities, helped uncover liver physiology and biochemistry. While these whole organ techniques were extremely informative, further study required isolation of the viable hepatocytes [17, 18]. Availability of hepatocytes furthered mechanistic studies, and provided a cell source for in vivo hepatocyte transplantation studies. However, despite these advances, new challenges were apparent, as isolated hepatocytes were unable to be cultivated in vitro for more than twenty-four hours using traditional seeding on tissue culture-treated plastic.

### Development of long term primary hepatocyte culture

#### Hepatocyte culture

**Fig. 2:**
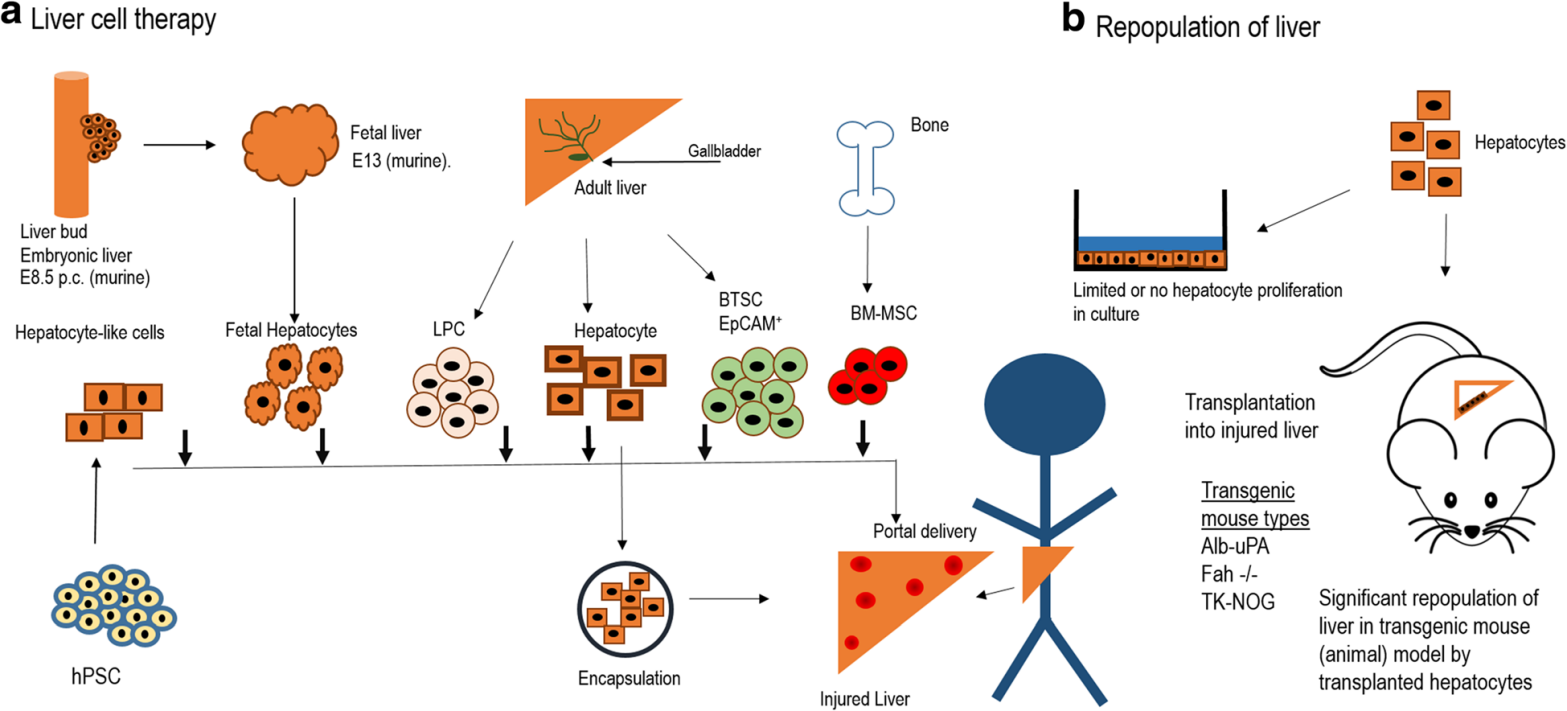
Liver cell therapy and liver repopulation. **a** The various types of liver cell therapies. Liver cell therapy utilizes a wide range of cells, from different stages of liver development and different tissue types, to treat acute or chronic liver disease. The liver develops from the liver bud (embryonic liver, E8.5) to form the fetal liver (fetal hepatocytes), neonatal liver, followed by adult liver (hepatocytes, liver progenitor cells, (LPC), biliary tree stem cells (BTSC)). Adult bone marrow (bone marrow-derived mesenchymal cells(BM-MSC), and other bone marrow cells) are also used as a cell source. Human pluripotent stem cells (hPSC) are used for differentiation towards hepatocyte-like cells. In encapsulation, shown below, therapeutic cells are placed within microcapsules to improve cell viability upon transplantation by protecting therapeutic cells. Delivery to the liver is often via the portal vein. **b** Liver Repopulation. Liver repopulation is an experimental procedure performed in transgenic mice. Activation of transgene in the liver injures or kills endogenous hepatocytes. Because of the regenerative capacity of the liver, the remaining injured cells would normally replicate within the liver to replace, or repopulate any empty areas. Because the endogenous hepatocytes are injured, they are unable to repopulate the liver. At the same time, if healthy hepatocytes are injected into the spleen, they will outcompete the native hepatocytes and will repopulate the liver. This assay can be used to assess the regenerative function of adult hepatocytes. Furthermore, it can be used to create a chimeric mouse with new hepatocytes, which may carry a disease causing gene and be used for disease modeling. Finally, if the host mice are both transgenic and immunodeficient, the animals can be used to bear human hepatocytes within the mouse liver, to create a human in-liver mouse. This can be used for disease modeling or testing hepatotoxicity or drug metabolism of human hepatocytes in mice. These transgenic animals can be used to expand primary hepatocytes, which typically don’t expand in culture

Although isolated hepatocytes were initially the mainstay of basic liver studies, the inability to cultivate hepatocytes long term, with physiological functions, limited potential applications (Fig. 2). Initial investigation of primary hepatocytes, employing cell adhesion to tissue culture plastic, resulted in a flat, rather than cuboidal, morphology, characterized by dedifferentiation. Based on initial findings, scientists developed co-cultivation approaches based upon extracellular matrix composition, with a focus for maintaining liver morphology and functions long term [19–21]. The culture of primary rat hepatocytes in a double gel, or sandwich, configuration, was the first experimental culture system displaying physiological functions for greater than 3 weeks [22]. In these seminal studies, the measurement of albumin production rate was utilized as a metric for liver synthetic function, and measured ~5 μg/h per 2 million plated hepatocytes. Urea, a product of ammonia metabolism related to hepatocyte catabolism of proteins and amino acids, measured ~4 μg/h per 2 million cells after 2 weeks of culture. The analysis of the functionality of liver specific cytochrome P450 (CYP) enzymes, which participate in metabolism of drugs and toxic compounds, showed increased activity. In addition, hepatocytes excrete bile across the apical domain of the cell membrane. Early studies highlighted the detection of bile canaliculi proteins and hepatocyte bile secretions in the double gel culture systems. These seminal studies opened up further areas of in vitro investigation, regarding the hepatocyte’s biochemical and metabolic responses to oxygen, lipids, and plasma exposure in the setting of bioartificial design [23–26].

In vitro hepatocellular organization, as a function of microenvironment, has been investigated with careful application of engineering tools and technologies. When cultivated on biomechanically soft surfaces, hepatocytes form three dimensional clusters, or aggregates. Interestingly, these aggregates also lead to stable hepatocyte functions and are an alternate culture configuration [27, 28]. Studies demonstrate that the advantages of aggregate culture, synonymous with spheroid or organoid culture, include increased cell-cell homotypic interactions, and ease of manipulation of tissue units. The disadvantages of aggregate culture include transport limitations, cellular heterogeneity, and lack of cord like liver microstructure. Thus each culture system can be chosen based on the question being asked and tailored to the desired application [29].

In parallel with development of culture systems, scientists explored the integration of engineered biomaterials with hepatocyte culture. Investigation into the science behind hepatocyte morphology and function demonstrated that aggregate size, scaffold topography, mechanochemical interactions, and ligand presentation distinctly modulate hepatospecific functions [30–33]. To model multicellular cords in the liver, bioengineers developed multilayering approaches based on thin polyelectrolyte films [34, 35] which maintain hepatocellular functions. Collectively, these studies highlighted the complex effects of extracellular matrix on hepatocyte morphology and function, and led to the development of key hepatocyte culture and biomaterials design principles.

#### Hepatocyte coculture

Another avenue of hepatocyte cell culture research focused upon the recapitulation of the normal hepatocellular milieu by examining cell-cell interactions (Fig. 1). The functional unit of the liver is the hepatic sinusoid, which is comprised of liver capillaries or sinusoids lined by specialized, liver sinusoidal endothelial cells, which contain unique pores (fenestrations) that facilitate material exchange. Between the endothelial lining and the microvilli-bearing hepatocyte, is an extracellular space known as the Space of Disse, and specialized supporting cells known as hepatic stellate cells. Hepatic stellate cells are present on the basal (sinusoidal facing) surface of the hepatocytes. Bile canaliculi combine to form intrahepatic ducts, lined by hepatobiliary duct cells, which carry the bile produced by the hepatocytes. Not surprisingly, initial hepatic coculture studies demonstrated stable hepatic functions [36, 37]. Building on this, three dimensional coculture systems sprang forth which relied specifically on both aggregate hepatocyte culture and interactions between hepatocytes and liver sinusoidal endothelial cells [38]. A third coculture approach utilized a transwell configuration to mimic not only the appropriate sinusoid cell types, but also the actual sinusoid geometry [39]. The choice of coculture cell type and configuration was found to differentially modulate hepatic specific functions [40]. In fact, cocultivation of hepatocytes and liver sinusoidal endothelial cells, in specific culture orientations, led to 10 times higher albumin function and 20 times higher CYP 1A1/2 cytochrome activity compared to controls [41]. interactions between hepatocytes and endothelial cells interactions have been shown be mediated by both growth factors [38] and intercellular amino acid transport [42].

With the success of cocultures but the need for enhanced cellular organization, engineers began to apply microfabrication technology for high level control of cellular level processes, such as cell-cell, nonparenchymal cell-cell (heterotypic), and cell-matrix interactions. These approaches served to preserve, modulate and enhance hepatocyte-specific functions [43, 44]. Further, these new techniques enabled further investigations of the science behind cellular interactions, which was possible through precise engineering and cell surface modification [45, 46]. These seminal studies primarily focused on fibroblast cocultivation, particularly with the specialized, NIH 3T3-J2 cell line, which uniquely boosts hepatic specific functions through cell-cell contacts with potential mechanisms explored [47, 48]. These microfabricated coculture systems have shown a great deal of utility in disease modelling [49, 50] and therapeutic applications [51].

### Bioartificial liver

**Fig. 3:**
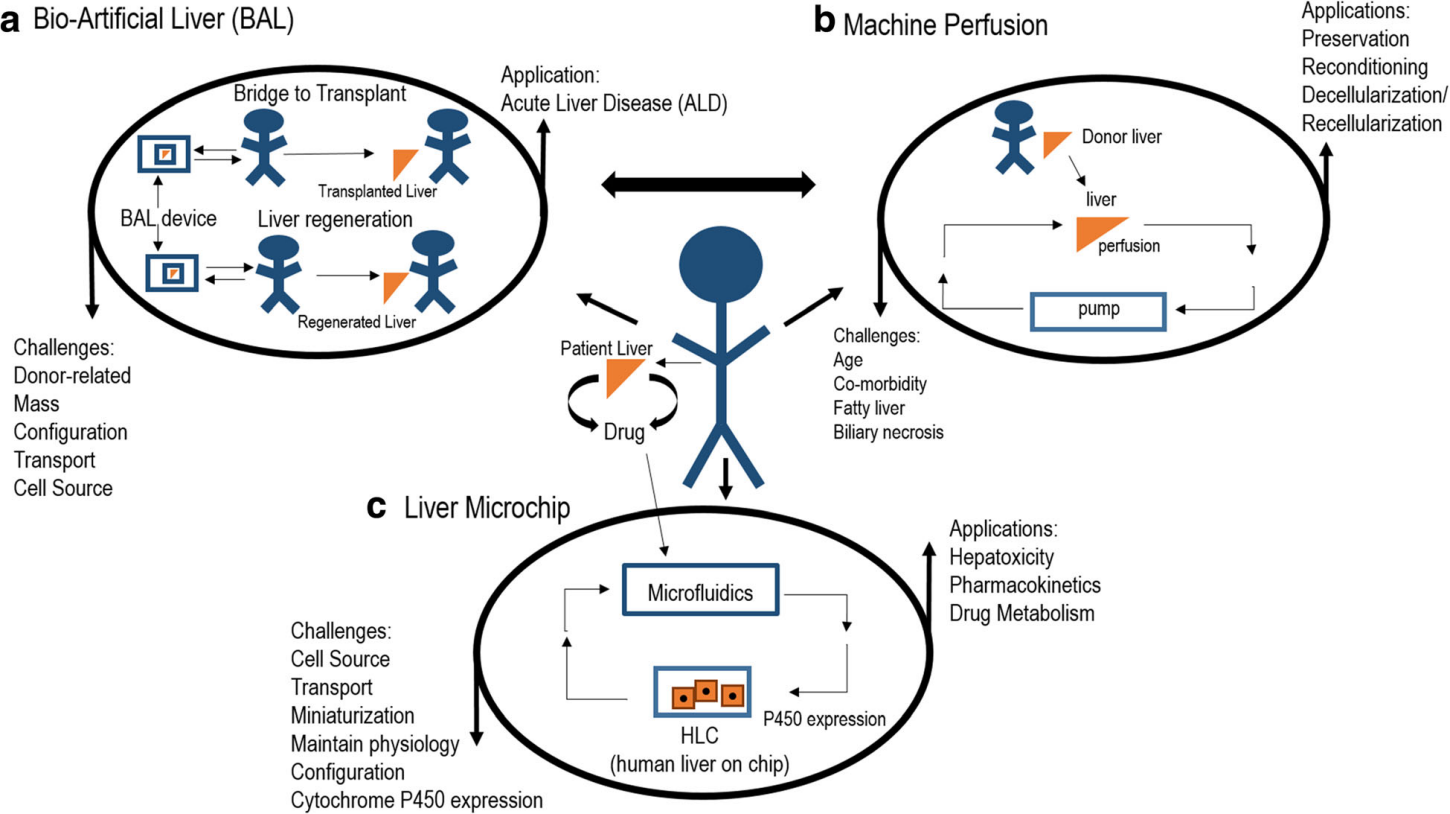
Engineered liver devices. **a** Bioartificial liver. Engineered liver devices are at different scales and have a wide range of applications. The Bioartificial Liver (BAL) is a bioreactor system which bears hepatocytes in a variety of formats (hollow fiber vs. spheroid vs. monolayer culture). A large number of hepatocytes, approximately 10% of the adult liver, are needed to provide appropriate level of functions. Typically, the BAL is used for acute liver disease. In this case, it can be used a bridge to transplant, or as a way to regenerate acutely injured liver. The main challenges and applications are as shown. **b** Machine Perfusion. This is a technique used for several applications in animal models. The whole liver is connected to the perfusion device and perfusate is oxygenated and pumped to perfuse the whole liver under hypothermic or normothermic conditions. The technique is used to preserve organs after harvest, as opposed to storage of organs without flow in organ preservation solution. Machine perfusion is also used to condition marginal livers, for example by adding medium components to reverse fatty liver disease in a donor liver. Finally, machine perfusion can be used to understand complex, whole liver metabolic functions by measuring metabolites at inlet and outlet of the device under various experimental conditions. The main challenges and applications are as shown. **c** Hepatocyte Microdevices. This is a technique in which the hepatocytes are placed within miniature microfabricated devices so that they display physiological functions. Both animal and human liver on a chip applications are possible, and are valuable for assessing hepatotoxicity, drug metabolism, and pharmacokinetics, in the setting of drug discovery. These devices can potentially replace animals in the drug discovery pipeline. Patient-specific hepatocytes can be used to understand how genetic variations effect drug metabolism. Multiple cell types can be used in a circuit to better model the human body. The main challenges and applications are as shown

Acute hepatic failure is accompanied by the loss of hepatic specific functions and disruption of basic liver physiology leading to complications and eventually death (Fig. 3). As early as the 1950’s, scientists experimented with a bioartificial liver (BAL) for support of hepatic failure [52]. The first BAL consisted of cross hemodialysis between blood from a living dog and an encephalopathic dog. Subsequently, a cirrhotic patient with hepatic coma was cured by a similar system [52]. Modern BAL systems contain hepatocytes in an extracorporeal support system, and design parameters include device structure and design, species of cell (human or xenogeneic), cell type (primary vs. transformed vs. cancer line), hepatocyte configuration (adherent vs. immobilization vs. encapsulation), cell mass, perfusate contents (whole blood versus plasma) and perfusion duration [53–56]. As stated above, the mass of hepatocytes required is 10% of liver weight [7]. Other core engineering concepts include cryopreservation of hepatocytes and of BAL devices [57–59], mass transport within the device, blood constituents, and their effects on liver function [25, 60], and integration of microfabrication technology with bioreactor design [61]. The studies in patients have been limited, but positive. For example, a BAL comprised of primary porcine hepatocytes demonstrated no toxic effects and functioned as a bridge to transplantation when used intermittently in patients [62]. A prospective, randomized, multicenter clinical trial of microcarrier attached, porcine hepatocytes demonstrated no difference between study groups, but again demonstrated safety, in addition to improvements in patient subsets of fulminant/sub-fulminant failure [63]. Finally, a promising recent preclinical BAL utilizing pig hepatic spheroids in a porcine acute liver failure model demonstrated that BAL support improved survival [64]. These studies indicate the BAL is a safe and promising temporary support for acute liver failure, and continued re-engineering of design, as well as randomized, double blinded clinical studies, are needed to demonstrate efficacy.

### Hepatocyte transplantation

The establishment of techniques for hepatocyte isolation, together with the development of animal models of liver failure, led to experimental hepatocyte transplantation (HCT). A detailed analysis of HCT has been reviewed elsewhere [65, 66]. HCT has utility for both temporary support for acute liver failure, and potentially for long term functional replacement for chronic liver diseases (Fig. 2). Initial attempts at liver transplantation of partial autografts, or portions of the liver, demonstrated lack of vascularization, cellular death and scarring [67, 68]. In contrast, initial studies of HCT demonstrated feasibility and therapeutic effect when delivered via portal vein in Gunn rats, which lack the liver enzyme uridine diphosphate glucuronyl transferase [69]. These Gunn rats function as a model of Crigler Najjar Syndrome, a rare congenital, autosomal recessive disorder of bilirubin metabolism. Positive benefits were identified not only in the Gunn rat model, but also in other acute liver failure models [70]. While initial studies employed intraportal and intraperitoneal injection, intrasplenic HCT also developed as an alternative transplant location [71]. The mechanism by which HCT worked was by the manifestation of liver nodules by transplanted hepatocytes, with maintained cellular ultrastructure [72]. Further characterization of these nodules was possible with suppression of endogenous hepatocyte replication by retrorsine after HCT and partial hepatectomy. These studies quantified the growth kinetics of donor hepatocyte cell clusters as they form intrahepatic nodules. 20–50 cells per cluster were present after two weeks, 100 cells per cluster were present at 1 month, and up to several thousand cell per cluster (representing 40–60% of hepatic mass) were present at 2 months. Further the investigation of regeneration demonstrated that soluble factors from supernatants of hepatocyte cultures can reverse liver injury, indicating complex and compensatory liver regeneration mechanisms [73]. To improve HCT for long term function, investigators attached hepatocytes to microcarriers [74], which could then be encapsulated [75, 76], as another HCT approach for liver disease. The identification and development of transgenic mouse disease models furthered the field, including the Gunn Rat, and the analbuminemic (albumin deficient) mice for measuring albumin production solely from transplanted hepatocytes, and immunodeficient animals for human HCT in rodents. Biomaterials and chemical technologies have also been used to improve HCT. Encapsulation is a technique in which cell mass can be incorporated into a semipermeable biopolymer droplet, often with a protective coating [77]. Encapsulation of hepatocytes [78], enables immunoisolation and intraabdominal cell transplantation [79], with maintained hepatocellular functions [80, 81].

Based on strong preclinical research, several clinical trials of HCT have been completed, indicating favourable regulatory approval and safety. Patients with acute liver failure benefit from hepatocyte infusions that provide weeks to months of support, as borne out by studies of auxiliary liver transplant in acute liver failure [7]. On the other hand, HCT in end stage liver disease, is likely hindered by the underlying pathology, including portal hypertension and highly abnormal tissue architecture. These cell transplantation studies demonstrate that efficient cellular delivery and engraftment are essential to improved therapeutic outcomes. The wide range of transplant locations used include intraperitoneal, intrasplenic, and intraportal and may affect cell engraftment [82].

### Mouse liver repopulation with hepatocytes

A series of studies with transgenic mice led to detailed analysis of hepatocyte repopulation ability within the liver of transgenic hosts (Fig. 2). Albumin-uroplasminogen activator (Alb-uPA) mice, which bear a hepatotoxic (uPA) gene, was the first model used [83]. Homozygous Alb-uPA mice died due to neonatal hepatocellular injury, while hemizygote Alb-uPA mice displayed hepatic nodules with liver function due to transgene inactivation. Each hepatic nodule was clonal, derived from a single hepatocyte lacking Alb-uPA gene expression. Transplanted adult hepatocytes in neonatal (1–2 week old) Alb-uPA mice demonstrated liver repopulation capacity at 5–7 weeks [84]. In this study, transgenic (genetically marked) hepatocytes were transplanted in the spleen and identified in in excised liver tissues. Liver nodules of the donor hepatocytes were generated at the expense of Alb-uPA expressing, injured, endogenous hepatocytes. Approximately twelve population doublings (~80% liver replacement) occurred per transplanted hepatocyte. In comparison, one or two doublings occur after hepatectomy/regeneration, and less than twenty-eight doublings replace total mouse hepatocyte mass in mice. This indicates the enormous repopulation capacity of primary hepatocytes.

Further investigation of repopulation was enabled by continued development of transgenic models, one of which was the Fah −/− mouse, a model of hereditary tyrosinemia type 1 [85, 86]. Fah −/− mice normally die from neonatal, hepatocellular injury, due to fumaryl acetoacetate hydrolase (Fah) deficiency, but are rescued with NTBC (2-(2-nitro-4-trifluro-methylbenzoyl)-1, 3-cyclohexanedione), which blocks tyrosine metabolism. NTBC treatment enables Fah −/− adult mice to maintain health, but when NTBC is withdrawn, adults die of liver failure in two months. In this model, when Fah + wildtype hepatocytes are transplanted intrasplenically and NTBC is withdrawn, they outcompete the endogenous Fah −/− hepatocytes and repopulate the Fah −/− liver. Moreover, NTBC administration suppresses the repopulation effect. In these seminal studies, the minimum number of cells required for liver repopulation was 1000 cells, and repopulation occurred between 4 and 8 weeks after transplantation. In fact, the repopulation capacity of hepatocytes was shown to be sixty-nine and eighty-six doublings in the Fah −/− model [86], enough repopulations to account for several livers. Furthermore, Fah gene delivery in these Fah −/− mice resulted in Fah + hepatocyte repopulating nodules. The crossing of this mouse with immunodeficient mice resulted in the FRG (Fah −/− Rag 2 −/− Il2rg −/− mouse). In FRG mice, human hepatocyte repopulation was demonstrated, leading to generate chimeric, human in mouse (HIM) livers [87]. In these HIM livers, human albumin serum levels and P450 enzymatic activity were found to correlate with percent of human hepatocyte repopulation. Other transgenic models were developed and furthered our understanding of liver repopulation including the TK-NOG (albumin thymidine kinase transgenic-NOD-SCID-interleukin common gamma knockout) mouse [88], and the AFC8 (FKBP-Caspase 8 gene supported by albumin promoter) mouse. Thus far, these HIM liver models are powerful tools for the study of human drug metabolism [89], hepatitis [90], malaria [91] and familial hypercholesterolemia [92], amongst others. Most recently, these transgenic systems have been applied to generate large animal (swine) models with repopulated livers [93].

Not surprisingly, these HIM models have been commercialized for these numerous applications. Yecuris (Tualatin, OR, www.yecuris.com) was founded in 2007 to commercialize the FRG technology. Hera Bio Labs (Lexington, KY, http://www.herabiolabs.com) founded in 2015, performs precision toxicology services with gene edited animal models, and is currently developing rat analogs of transgenic mouse liver repopulation models. IMODI (France, http://www.imodi-cancer.org) is a French consortium which uses the TK-NOG liver humanized model, for generating human specific profiles of chemotherapeutics. KMT Hepatrhc (Edmonton, Alberta CA, http://www.kmthepatech.com) developed the KMT Mouse™, uses the uPA+/+/SCID mouse to generate a chimeric mouse with a humanized liver. These preclinical HIM tools are being widely utilized for drug discovery, development and preclinical.

### Cell-based liver therapies

The growth in adult and pluripotent stem cell (PSC) biology and the boom in cell therapies has reinvigorated the field of liver cellular therapy. Identifying a robust hepatocyte cell source is a significant bioengineering challenge within the field of liver cell and tissue therapy. A wide range of cell types in preclinical and clinical models have thus far been utilized (adult hepatocytes, fetal hepatocytes, bone marrow-derived cells, adult stem/progenitor cells) (Fig. 2). Donor variability and marginal donor sources are major impediments to obtaining transplantable hepatocytes. Primary hepatocytes are needed in large quantities, and do not replicate in vitro. However, recent studies demonstrate appreciable in vitro hepatocyte expansion [94–96], but these approaches have not yet been adopted for widespread use. Another solution is the immortalization of primary hepatocytes which confers proliferative capability, via conditional or constitutive upregulation of immortalization genes [97, 98]. Although these immortalized hepatocytes exhibit unlimited replication and represent a uniform cell source, they exhibit decreased hepatic functions and carry increased risks for tumorigenesis [98–100]. Xenogeneic (porcine) hepatocytes are an additional hepatocyte cell source. However, differences between physiological functions and responsiveness of porcine hepatocytes in a human environment is a major concern [101], although they also represent an endless supply of isogenic and uniform hepatocytes. Further, these hepatocytes can be either genetically engineered [102] or encapsulated [76], to evade the immune system. Fetal liver progenitor cells (hepatoblasts) have also emerged as an alternative to primary hepatocytes given their proliferative capacity and predisposition to develop into both hepatocytes and cholangiocytes (Fig. 2) [103]. They have been used to repopulate the liver of immunosuppressed rats and mice [104], and used in clinical studies. However, to be valuable as a cell source, these fetal progenitor cells need to be uniform with respect to age, proliferative capacity, and donor matching.

### Adult liver stem/progenitor cells

Adult, resident, liver stem/progenitor cells (LSC) are also candidates for liver cell therapy in preclinical and clinical studies (Fig. 2). Normally, quiescent, self-renewing adult stem cells reside in tissues and play a key role in replenishing tissues and maintaining tissue homeostasis, in tissues like the bone marrow, intestine, and skin. In acute liver injury, hepatocytes contribute to normal liver homeostasis through replication, functioning like a stem cell. However, in chronic injury, particularly in rat and in human liver, not only do hepatocytes replenish liver tissue, but also LSC may play a greater role. LSC take the form of bipotent, small, oval shaped, progenitor cells which express cytokeratins and give rise to cells from the hepatic and biliary lineage [105]. Foxl1 has been shown to be a marker in this bipotent stem cell population [106]. Further, the cells lining the intrahepatic and extrahepatic duct contain Sox9 positive progenitor cells, and contribute to bile duct and hepatocyte homeostasis as shown by lineage tracing in mouse models [107]. Another key LSC population is biliary tree stem cells (BTSC), which are quiescent, self-renewing stem cells that reside in the peribilliary glands, and may give rise to hepatic/stem progenitor cells [108]. Clinical liver cell therapy studies with these BTSC, marked by Lgr5 (Leucine-rich repeat-containing G-protein coupled receptor 5), EpCAM (Epithelial cell adhesion molecule), and pluripotency genes like Oct4, Sox2, are in progress. Collectively, these studies of LSC subtypes demonstrate several investigators contributions to animal models of hepatocellular injury, the subtleties of the responses in the different models, the difficulty of tracking and analyzing small numbers of multipotent cells, and the promise for liver therapies [108].

### Pluripotent stem cell (PSC) technologies

**Fig. 4:**
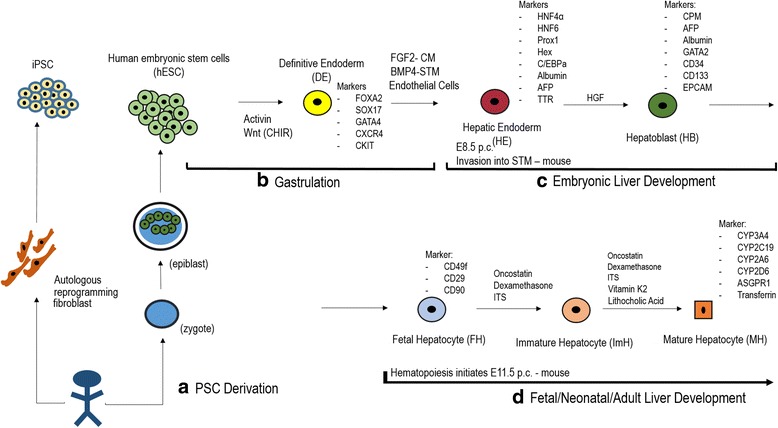
Mature hepatocyte differentiation from human pluripotent stem cells (hPSC). **a** Human pluripotent stem cell (hPSC)-derivation. hPSC either can be human embryonic stem cells (hESC) or human induced pluripotent stem cells (hiPSC). hESC are derived from the fertilized human embryo at the blastocyst/epiblast stage of development. hiPSC are derived from any cells of the patient, typically fibroblasts. Typically, four factors are activated within the fibroblasts to engineer iPSC, which are patient-specific. Cells are maintained in colonies with specialized serum-free medium and cultivated on basement membrane-resembling matrix. **b** Gastrulation. hPSC undergo similar signaling to what occurs during early development of the organism into germ layers, such as ectoderm, endoderm, and mesoderm. Activin and/or Wnt activate key pathways that induce primitive streak mesendoderm and endoderm from hPSC. The transcription factors and cell surface markers activated are as shown. **c** Embryonic liver development. hPSC-endoderm can form hepatic endoderm (HE) in the presence of growth factors (FGF2 from cardiac mesoderm (CM), BMP4 from septum transversum mesenchyme (STM), and cell-cell contacts with endothelial cells. These then activate HE specific markers. The HE cells delaminate out of the epithelium into the STM during this stage and form hepatoblasts in the presence of HGF. The cells from cords of hepatoblasts (markers as shown). The STM is obliterated. Markers are as shown. **d** Fetal, neonatal, and adult liver development. hPSC-hepatoblasts can be matured in the presence of maturating agents like Dexamethasone, Oncostatin, and Insulin, Selenium, and Transferrin (ITS). The result is fetal, neonatal, immature, and eventually, mature hepatocyte cells. These mature cells can potentially be used in a wide range of hepatic devices, basic studies, and cell therapy protocols

The advent of pluripotent stem cell (PSC) technologies has greatly accelerated the development of a self-renewable liver cell source (Fig. 4). The origins of PSC technologies began with the development of nuclear transfer techniques that enabled cloning of organisms, termed reproductive cloning. In seminal studies, the nucleus from a frog blastomere was transplanted into enucleated frog oocytes and generated early cleavage embryos [109], and building on this, an adult, somatic nucleus was reprogrammed to a pluripotent state [110]. A blastocyst generated by this reprogrammed, somatic nucleus was transplanted into a pseudopregnant mouse, giving rise to a clone with a genome of the donor nucleus [111]. These techniques enabled the production of cloned animals, and eventually, transgenic mice, and furthered our understanding of pluripotency. The next scientific contribution was the isolation of a pluripotent, self-renewing cell population derived from the inner cell mass of the mouse blastocyst [112, 113], called mouse embryonic stem cells (mESC). Demonstrating their pluripotency, mESC can give rise to all three germ layers in vitro and to a teratoma when transplanted ectopically in vivo. Further, when incorporated into chimeric blastocysts that are transplanted into pseudopregnant mice, the mESC genome can be passed through the germline to form new clones. Success of mESC derivation led to derivation of mESC from other species, including rat, cow, and pig. Similarly, human ESC were isolated from human embryos, normally discarded from in vitro fertilization centers and culture techniques were established [114]. Scientists then determined the culture conditions for ESC to self-renew and maintain pluripotency in vitro. Despite their potency, hESC brought about ethical issues because of their association with discarded human embryos.

The development of techniques to reprogram adult cells to PSC developed [115], as this would bypass ethical issues and improve patient-specific treatments (Fig. 4). In one approach, termed cell fusion, adult fibroblasts are fused with ESC, resulting in activation of pluripotency in the somatic nucleus. However, the resulting pluripotent cell is a heterokaryon [116]. In parallel, a potent, widely used approach developed for generating self-renewing, patient-specific PSC, from any donor cell type. Reprogramming of mature, somatic cells by introduction of 4 transcription factors simultaneously, resulted in induced pluripotent stem cells (iPSC). Since these iPSC could be generated without destroying human embryos, a new field within PSC biology [117, 118]. Nevertheless, both ESC and iPSC could be interchangeably used in PSC differentiation protocols.

The usefulness of PSC for liver differentiation is based upon fundamental studies of soluble factors and transcription factors that govern mouse liver organogenesis [119], as PSC in vitro follow highly regulated, developmental programs that normally occur in vivo. Liver differentiation protocols, based on these pathways, directed mouse ESC towards liver-like cells [120–122]. In these studies, stable transplantation of cells into liver tissue and enhanced survival of animals in liver disease models [123–125]. Further studies focused on the nature of the earliest progenitors of the liver, the definitive endoderm, from mouse and human PSC studies, about which little was known [125–129]. The study by Parashurama et al. [128] was one the first demonstrating that mESC-derived endoderm progenitor cells, upon transplantation, can give rise to three dimensional, vascularized tissues, and the study by Cho et al. [125] was the first to demonstrate a specific technique for rapid mESC-derived endoderm expansion. hPSC studies (hESC or hiPSC) used similar approaches [130] based upon original mouse differentiation protocols, with the creative use of small molecules [96] reprogramming approaches [131], and extracellular matrix systems. Not only could PSC be differentiated towards the liver lineage, but also other approaches were developed. Transdifferentiation, or directed reprogramming of hepatocytes from fibroblasts, was established using key liver specific transcription factors [115, 132]. Directed differentiation protocols resulting in liver differentiation demonstrated liver specific morphology and gene expression [130]. However, limitations have been the lack of fully mature hepatocyte functions, including albumin secretion, P450 activity, urea function, and the inability to fully repopulate the liver upon transplantation in transgenic liver injury models. Despite their lack of maturity, PSC-derived hepatic-like cells have successfully been employed in approaches to model liver diseases in vitro [133].

### Hepatotoxicity and engineered microdevices

Cell based systems are useful for applications requiring in vitro models that mimic liver functions (Fig. 3). Pharmaceuticals which mediate drug induced liver injury (DILI) are a major public health problem with heightened focus in recent academic and industrial research [134]. The liver is a central player in drug metabolism, and employs the Phase I system which is a mixed function oxidase system, including the P450 enzymes, and Phase II involving conjugation for improved solubility and drug excretion. Not surprisingly, hepatotoxicity is the number one reason that medications are withdrawn worldwide [135]. Traditional models of hepatotoxicity testing employs isolated microsomes which contain key detoxification enzymes, liver cancer cell lines, isolated primary hepatocytes, and liver slices [136]. However, several approaches applying bioengineering principles are in development to improve hepatotoxicity testing. Real commercially available products of engineered hepatocyte based systems are offered by several companies, including Regenemed (http://www.regenemed.com, San Diego, CA), InSphero (https://www.insphero.com, Schlieren, Switzerland), and Hepregen (http://www.hepregen.com, Medford, MA). These companies employ co-culture, plate based two dimensional, or three dimensional systems, characterized by various culture configurations composed of hepatocytes and nonparenchymal cells.

While these techniques can be considered static, more dynamic systems exist which employ cell culture, microfluidic technology, and bioreactor approaches. Flow-based systems which mimic oxygen and nutrient transport, and waste exchange, demonstrate improved cell culture parameters [137]. Cell Asic (Hayward, CA) [138, 139] uses microfabricated porous channels which function as artificial endothelial barriers to protect hepatocytes from shear effects with improved nutrient exchange. Similarly, a device by CN Bio Innovations Ltd. (http://cn-bio.com/cn-bio-launch, Oxfordshire, UK) pumps medium from a reservoir to a reaction chamber, which bears cocultured hepatocytes. Hurel (http://hurelcorp.com, Beverley Hills, CA) cocultures hepatocytes in microfluidic small scale cell culture analogs (μCCA). These μCCA can be integrated in microfluidic flow systems, with chips bearing other cell/tissue types, to better model whole body metabolism mediated by the liver. These devices show an in-vivo like metabolism in response to various drugs [140]. 3D printing approaches, which have the benefit of reduced cost and increased ability to generate layered systems, are being developed for a new generation of liver based devices. Organovo (http://organovo.com, San Diego, CA), employs 3D printing with devices bearing tissue-tissue interfaces and spatio-temporal diffusion of bio-chemicals, within a mechanically robust micro-environment [141]. Another innovation in drug metabolism studies has been at the level of cell source. The HepRG cell line is a bipotent liver cell line that, when differentiated further, better mimics hepatocytes, compared to comparable cancer cell lines. It offers uniformity of gene expression and drug metabolism, and functions as a key alternative [142, 143].

### Engineering considerations of hepatocytes within devices

Whether hepatocytes are cultivated within bioreactors or microdevices, in vitro bioprocessing of mature hepatocytes involves considerations of cell seeding, hepatocellular, and extracellular matrix configuration (Fig. 3). Viability, morphology, and function are major considerations within the microenvironment of these devices. In addition to monitoring changes in temperature and pH, growth factors, oxygen, and nutrients, are essential for maintained hepatocellular functions, as is removal of waste products. Flow-based systems improved physiological modeling of liver functions, but associated biomechanical forces within these engineered microenvironments will affect cells. Hydrodynamic shear stress associated with spinning bioreactors and shear stress associated with capillary motion of cells in liver cell microchips naturally impacts cell behavior. Preclinical models of the BAL have determined how flow affects primary rat hepatocytes (cocultured with 3 T3) functions [144]in a microchannel bioreactor system. Shear stress calculations showed that low wall shear stress for the bioreactor (0.01 to 0.33 dyn/cm^2^) hepatocyte function measured in albumin and urea synthesis rates, were 2.6 to 1.9 times, respectively, greater than those at higher wall stresses (5 to 21 dyn/cm^2^). A follow up study validated the detrimental effects of shear stress on hepatic function, while developing grooved substrates that protect hepatocytes from shear under high flow/oxygen delivery conditions [61]. These studies highlighted shear stress effects but lacked the cellular content and geometry that is present in the liver sinusoid. Du et al. [145] created a model of the liver sinusoid, complete with a fluid channel for flow lined by liver sinusoidal endothelial cells (LSEC) and Kupfer cells lying on a porous membrane. These pores lead to a second channel with primary hepatic stellate cells and primary hepatocytes. These studies demonstrated that shear flow (0.1–0.5 dyn/cm^2^) enhanced albumin, HGF secretion, as well as drug metabolism, but not urea secretion. However, oxygen transport was not modeled in this study. Overall, micro-engineered organ on a chip technology that integrates defined 3D microarchitecture, hepatocytes, microscale interactions, and microfluidics, report enhanced liver functions in the presence of oxygen and shear flow.

### Decellularized liver grafts

A new approach in liver regenerative medicine is generating three dimensional tissue with a decellularized, native liver bioscaffold, that can be re-seeded with appropriate parenchymal and nonparenchymal cells (Fig. 3). This whole organ approach may enable scientists to salvage marginal livers, or perhaps even xenogeneic livers for therapeutic use. Although decellularization has been used since the 1980’s [146], the first report of whole organ decellularization and recellularization resulted in a functional heart and opened up a new field in bioengineering and medicine [147]. Using similar techniques, scientists generated the first decellularized, and recellularized liver [148]. Here, ischemic liver decellularization with perfusion of sodium dodecyl sulfate (SDS) detergent preserved the chemical composition and structure, with structurally intact vessels, and bile ducts, and was recellularized with hepatocytes as well as microvascular endothelial cells under perfusion. The recellularized graft was transplanted for eight hours in vivo, perfused ex vivo for twenty hours, and demonstrated mature liver functions. Follow up studies demonstrated multistep cell seeding with proliferative hepatocytes, the presence of the biliary tree, a milder decellularization cocktail, the use of a cryopreserved, rather than ischemic, donor liver [149], and further process improvements [150, 151]. In bringing the approach to clinical scale, pig livers have been processed in a similar way [152]. These studies point towards success in the preclinical small and large animal studies, and fundamental limitations, such as seeding and in vivo survival, that are actively being addressed.

## Conclusions

In this review, we summarize the history and key publications within liver regenerative medicine. We summarize seminal studies in areas as diverse as liver perfusion and hepatocyte isolation, liver regeneration, bioartificial liver, liver transplantation, and cell therapies. These subjects have in part forged the field liver regenerative medicine. The largest discriminating factor in liver regenerative medicine is the shear mass of the liver, as it is a solid organ with ~2 × 10^11^ cells in a 70 kg male. Its vast size together with complex hepatocellular functions, including detoxification, whole body metabolism, digestion, and protein synthesis, naturally constrain in vitro models and therapeutic solutions. Below we analyze aspects of liver regenerative medicine some future areas of growth.

In terms of liver transplantation, the lack of donor livers has focused attention upon increasing donor pool through advancing living donor related transplantation, reconditioning marginal livers using machine perfusion, and whole organ decellularization. We speculate that improvement in this area could be achieved, conceptually, by combining transplantation technology, with liver regeneration fundamentals, and organ preservation technology. If donor tissue can be divided surgically into smaller transplantable units, could make several hepatectomized transplants available. If this hepatectomized transplant can be appropriately anastamosed to the hepatobiliary ducts, and both the portal and the systemic circulatory systems in a matched organ transplant recipient, then more transplants from an initial donor organ might be available. Approximately 10% of liver mass may be needed to maintain liver functions. Here, perhaps improved knowledge of liver regeneration could be used to grow the miniature transplant in the patient. The other transplantable units could be maintained through storage techniques and transplanted either at the same time in matching patients or at a future time. Further, perhaps transplantable units could be regenerated ex vivo using perfusion technology (see below). This approach could be used to salvage donor tissue, and potentially preserve tissue for multiple operations. As it stands now, donor limitations are a major problem and will continue to be.

A major area of potential, continued growth will likely be machine perfusion technology [153]. Storage under perfusion could reduce serial aspects of organ injury that occur during storage and transplantation [154]. Further, changes in donor pool, reflected by organs from older patients, donors with more concomitant disease, donors with steatohepatitis, and donors from non-heart beating donors, could all have a higher risk of delayed graft functions, [153]. and thus machine perfusion could address this problem [155]. Most liver transplant centers are not yet equipped with this technology. Opportunities in this area could be methods for making the process inexpensive and extending perfusion time. Furthermore, the roles of the perfusate type, oxygenation and temperature of perfusate, pressure versus flow based control of perfusate, length of perfusion, and assessment of metabolic parameters measured all are under investigation [153]. Machine perfusion has also been used to generate decellularized livers and potentially recellularized livers which is a likely growing application of this technology [156].

Although BAL technology for acute liver failure is again reaching the pre-clinical stage, there remain many challenges for clinical implementation. Here, the BAL could serve as a bridge to transplantation, or as a therapeutic intervention to improve symptoms. From a practical point of view, if a patient is diagnosed with acute liver failure (ALF) in a community hospital, rapid hepatocellular injury and subsequent encephalopathy would occur on a time scale of a week (hyperacute) to a month (subacute) [157]. Therefore, the patient would likely need to be sent to a liver specialty center and receive a BAL within days of diagnosis. The greatest limitation here is that a large cell mass, approximately 2 × 10^10^ functional hepatocytes, would need to be readily available. In the Glorioso et al. study [158], allogeneic porcine hepatocytes were used, and each swine that received BAL therapy required one swine donor liver to generate the high density hepatocyte culture within the BAL. If high density hepatocyte spheroid cultures can be rapidly deployed within 24 h as they were in the Glorioso et al. study, then perhaps this approach can be utilized clinically, because it fits with time in which patient’s with ALF develop symptoms. Probably porcine hepatocytes, or even better, human hepatocytes could be used in this BAL approach. Obtaining a large number of human hepatocytes in such a short amount of time would be problematic. However, liver repopulation has been accomplished in mice, rats, rabbits, and pigs [93]. It may be possible to also repopulate human hepatocytes in immunodeficient large animals as is done in mouse. If these animals, or the cells within them can be transported rapidly, it would be possible to obtain a large amount of hepatocytes that would be needed for a BAL to function.

There remains to be potentially valuable contributions for cellular therapies and stem cells in liver regenerative medicine. An effective use of HCT is acute liver failure. A major impediment is large numbers of an allogeneic hepatocyte cell source that would be required, and associated immunosuppression. As mentioned above, the only way to expand human hepatocytes to large quantities is with in vivo liver repopulation within immunodeficient, transgenic large animals. However, human hepatocytes would have to be recovered without any associated pig antigens, and at this time it is not 100% clear if this is possible. Genetically modified pigs, perhaps without hepatocyte MHC I or without cell surface carbohydrates that induce a hyperacute immune reaction, could also be potentially be used as a source of hepatocytes for human transplantation. However, associated immunosuppression would be required. The advantage of this approach compared to the BAL for acute liver failure would be cost, ease of application, and the fact that human hepatocytes from the same swine donor liver could potentially be used for another patient simultaneously.

hPSC are promising because they are self-renewable, and thus hPSC-based approaches to generate mature hepatocytes or mature liver tissue are advantageous. This would impact several major fields within liver regenerative medicine. As a central cell source for devices, like the BAL and human liver on a chip, and a source for hepatocyte cell therapy, this would be a major achievement in liver regenerative medicine. New methods to differentiate hPSC to hepatocyte-like cells [159] or to improve maturation of hepatocytes are likely to be important to generating fully functional hepatocytes [160]. These types of studies will be a key development within liver regenerative medicine. The fact that several hepatocyte-based approaches have led to several commercialization efforts, indicate that there is an indeed a “market” for liver cells/tissue at a time when liver diseases are escalating. As scientists, engineers, and physicians continue to work together on creative solutions, we expect further development of new technologies that will advance the field for improved patient care of patients with liver disease.

## Abbreviations

Alb-uPA: Albumin-Uroplasminogen Activator
BTSC: Biliary tree stem cells
CLD: Chronic Liver Disease
CYP: Cytochrome P450 Enzymes
DILI: Drug induced liver injury
EpCAM: Epithelial cell adhesion molecule
FAH: Fumaryl acetoacetate hydrolase
FRG: (Fah −/− Rag 2 −/− Il2rg −/− mice
HCC: Hepatocellular Carcinoma
HCT: Hepatocyte cell transplantation
HIM: Human in mouse
iPSC: Induced pluripotent stem cells
Lgr5: Leucine-rich repeat-containing G-protein coupled receptor 5
LSC: Liver stem/progenitor cells
NAFLD: Non-alcoholic Fatty Liver Disease
NASH: Non-alcoholic Steatohepatitis
NTBC: (2-(2-nitro-4-trifluro-methylbenzoyl)-1, 3-cyclohexanedione)
PSC: Pluripotent stem cells
SDS: Sodium dodecyl sulfate
μCCA: Micro-fluidic small-scale cell culture analogs
